# A Randomized Controlled Trial of Two Corticosteroid Dosages for Relapsed Childhood Steroid-Sensitive Nephrotic Syndrome: The OPtimising dosE in Nephrotic Syndrome (OPEN) Trial Protocol

**DOI:** 10.1016/j.ekir.2026.107115

**Published:** 2026-10-02

**Authors:** Nicholas G. Larkins, Hugh McCarthy, Simon A. Carter, Siah Kim, Anna Francis, Rachael Kermond, Lilian Johnstone, Chanel Prestidge, Wendy Cook, Dawn Sheriff, Kylie-Anne Mallitt, Andrea K. Viecelli, Xin Teng, Chandana Guha, Martin Christian, Susan Samuel, Kelly Loffler, Rupali Saikia, Martin Howell, Germaine Wong, Allison Jaure, Armando Teixeira-Pinto, Jonathan C. Craig

**Affiliations:** 1 Department of Nephrology and Hypertension, Perth Children’s Hospital, Perth, Australia; 2 Division of Paediatrics, Medical School, University of Western Australia, Perth, Australia; 3 Nutrition and Health Innovation Research Institute, Edith Cowan University, Perth, Australia; 4 School of Public Health, University of Sydney, Sydney, New South Wales, Australia; 5 Department of Nephrology, Children’s Hospital at Westmead, Sydney, New South Wales, Australia; 6 Department of Nephrology, Royal Children’s Hospital, Melbourne, Victoria, Australia; 7 Department of Paediatrics, University of Melbourne, Melbourne, Victoria, Australia; 8 Population Innovation, Murdoch Children’s Research Institute, Melbourne, Victoria, Australia; 9 Department of Nephrology, Queensland Children’s Hospital, Brisbane, Queensland, Australia; 10 Department of Nephrology, Women’s and Children’s Hospital, Adelaide, South Australia, Australia; 11 Department of Nephrology, Monash Children’s Hospital, Melbourne, Victoria, Australia; 12 Department of Paediatrics, Monash University, Clayton, Victoria, Australia; 13 Department of Nephrology, Starship Children’s Hospital, Auckland, New Zealand; 14 Nephrotic Syndrome Trust, Bath, England; 15 Australasian Kidney Trials Network, University of Queensland, Brisbane, Queensland, Australia; 16 Centre for Kidney Research, Children’s Hospital Westmead, Sydney, New South Wales, Australia; 17 Department of Nephrology, Nottingham Children’s Hospital, Nottingham, United Kingdom; 18 Division of Nephrology, British Columbia Children’s Hospital, Vancouver, British Columbia, Canada; 19 Department of Paediatrics, University of British Columbia, Vancouver, British Columbia, Canada; 20 Flinders Clinical Trials, Flinders University, Adelaide, South Australia, Australia; 21 College of Medicine and Public Health, Flinders University, Adelaide, South Australia, Australia; 22 Leeder Centre for Health Policy Economics and Data, University of Sydney, Sydney, New South Wales, Australia

**Keywords:** adolescent, child, corticosteroids, nephrotic syndrome, prednisolone, randomized controlled trial

## Abstract

**Introduction:**

Steroid sensitive nephrotic syndrome (SSNS) is a relapsing-remitting condition treated with repeated courses of high-intensity corticosteroids. Corticosteroid-related adverse effects cause morbidity and reduce quality of life.

**Methods:**

The OPtimising dosE in Nephrotic syndrome (OPEN) trial is a multicenter, randomized, controlled, noninferiority trial comparing high-intensity with low-intensity corticosteroid treatment in children (aged 1–16 years) with relapsed SSNS. For induction, participants will receive prednisolone 60 mg/m^2^ (maximum 60 mg) or 30 mg/m^2^ (maximum 30 mg) daily until remission. A second randomization, conditional upon remission, allocates maintenance prednisolone 40 mg/m^2^ (maximum 40 mg) or 20 mg/m^2^ (maximum 20 mg) every other day for 4 weeks. All Australian and New Zealand tertiary pediatric nephrology centers will participate. Exclusion criteria include corticosteroid use within 14 days, familial nephrotic syndrome, or biopsy findings other than minimal change disease or focal segmental glomerulosclerosis; noncorticosteroid immunosuppression is permitted. The sample size is 330, assuming median times to remission and relapse of 9 and 100 days. The primary outcomes are time-to-remission (induction) and time-to-relapse (maintenance), analyzed as the difference in restricted mean survival time (28- and 270-day horizons) with noninferiority margins of 3 and 30 days, and power calculated assuming a true difference of zero. Randomization (1:1) will use site-stratified, variably sized permuted blocks generated by the central trial pharmacy; participants, clinicians, site pharmacies, investigators, and data analysts will be blinded.

**Discussion:**

The OPEN trial will define the safety and efficacy of low-intensity corticosteroid treatment in relapsed SSNS. If noninferiority is demonstrated, cumulative corticosteroid exposure could be halved.

**Trial registration:**

Australian New Zealand Clinical Trials Registry (ANZCTR) ACTRN12624001201561p. Prospectively registered on 1 October 2024.

## Graphical abstract

**Figure:**
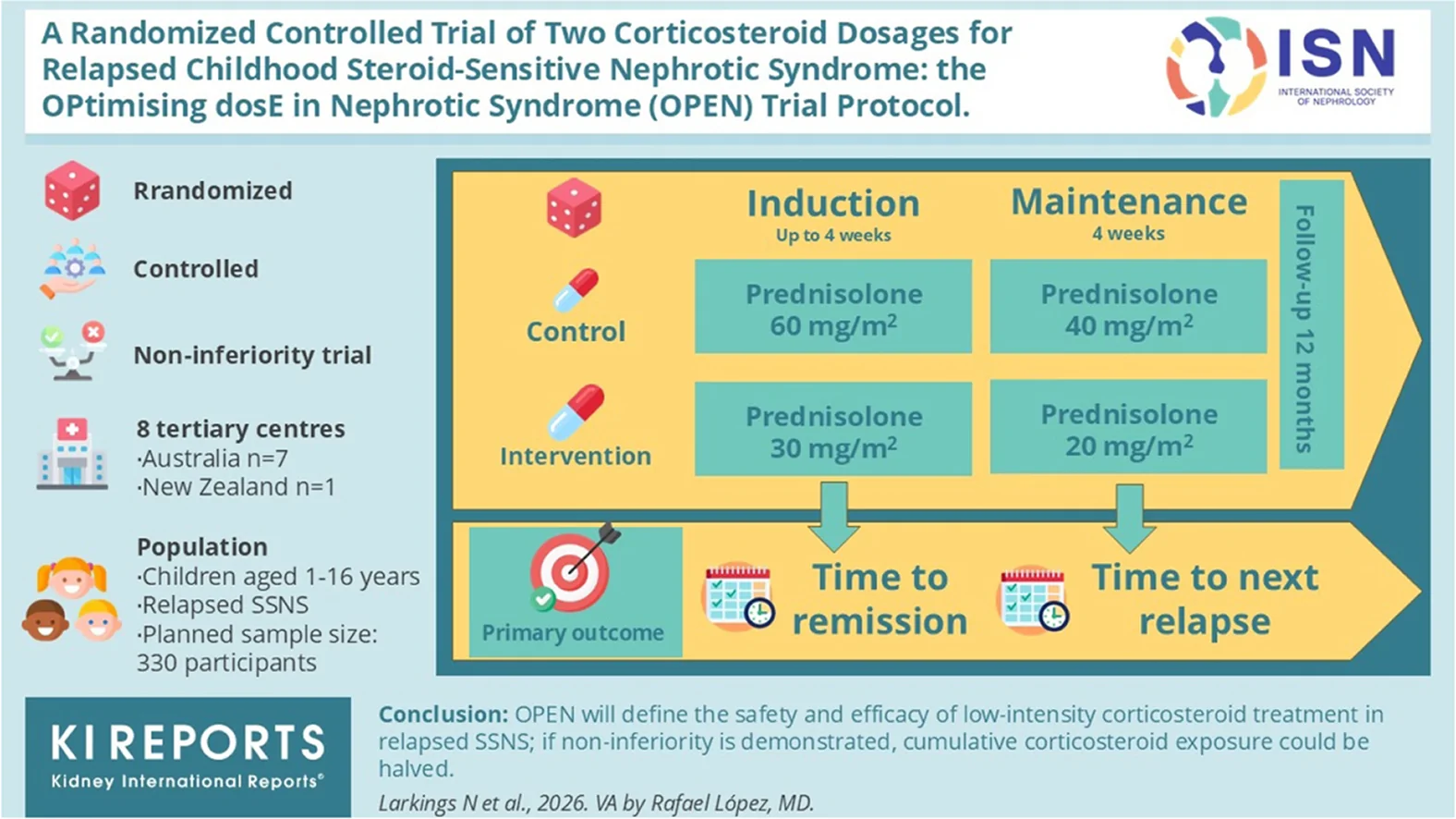

## Background and Rationale

Nephrotic syndrome, characterized by a triad of edema, proteinuria, and hypoalbuminemia, is an ultra-rare and orphan condition that mostly affects children. Although rare, it is one of the most common glomerular diseases in children, with an incidence of between 1 and 6 per 100,000 children per year, depending on geographic ancestry.^1^, ^2^ Spontaneous remission is rare and untreated children develop complications due to a failure of fluid and electrolyte homeostasis combined with a loss of proteins, via the urine, needed to prevent infection and thrombosis.^2^, ^3^ Childhood nephrotic syndrome is treated with corticosteroids to induce remission and is broadly stratified according to initial treatment response into steroid-sensitive (SSNS) and steroid-resistant disease (SRNS). SSNS (85% of children) comprises a group with immune-mediated disease, typically following a relapsing-remitting course,^4^, ^5^, ^6^ whereas SRNS (15% of children) represents either genetic, metabolic, or treatment-resistant immune-mediated disease.^2^ SSNS and immune-mediated SRNS result from autoimmune processes that damage podocytes or the slit diaphragm connecting them.^7^, ^8^

The use of high-dose corticosteroids to treat nephrotic syndrome revolutionized the outcomes in children with this condition. Before the introduction of antibiotics and corticosteroids, childhood nephrotic syndrome carried a mortality rate of 70%.^9^ However, modern treatment strategies have successfully reduced this to < 1%.^10^, ^11^ Although patient survival has improved, the disease burden remains substantial, with cumulative corticosteroid side effects a key contributor.^12^, ^13^, ^14^, ^15^, ^16^, ^17^ Treatment regimens for corticosteroid use in SSNS remain the same as those derived by expert consensus for the International Study of Kidney Disease in Children in the 1960s.^18^ Management of a relapse was 60 mg/m^2^ of prednisolone (or prednisone) daily to induce remission, defined by 3 consecutive days of trace or negative urine protein on dipstick, followed by a consolidation phase of 40 mg/m^2^ on alternate days for 4 weeks.^18^ The dose used is roughly twice that used to treat many other autoimmune diseases, and preliminary data suggest that this could be reduced without compromising efficacy.^19^, ^20^, ^21^, ^22^, ^23^ However, trials to-date have been small, often focusing exclusively on either the induction or maintenance phase and have generally excluded children with frequently relapsing disease or those on concurrent corticosteroid-sparing agents.

### Objectives

The OPEN trial aims to answer 2 questions for children with a relapse of SSNS. First, is low-intensity corticosteroid treatment equivalent to current standard of care to induce remission after relapse? Second, is low-intensity corticosteroid treatment equivalent to current standard of care to maintain remission after induction treatment? We hypothesize that low-intensity corticosteroid treatment will be noninferior to high-intensity corticosteroid treatment with respect to both induction phase and maintenance phase.

### Trial Design

The OPEN is a randomized, parallel-group, blinded, noninferiority trial. Participants will be randomized 1:1 to receive the intervention (low-intensity corticosteroid treatment) or the control (high-intensity corticosteroid treatment; Figure 1).

**Figure 1:**
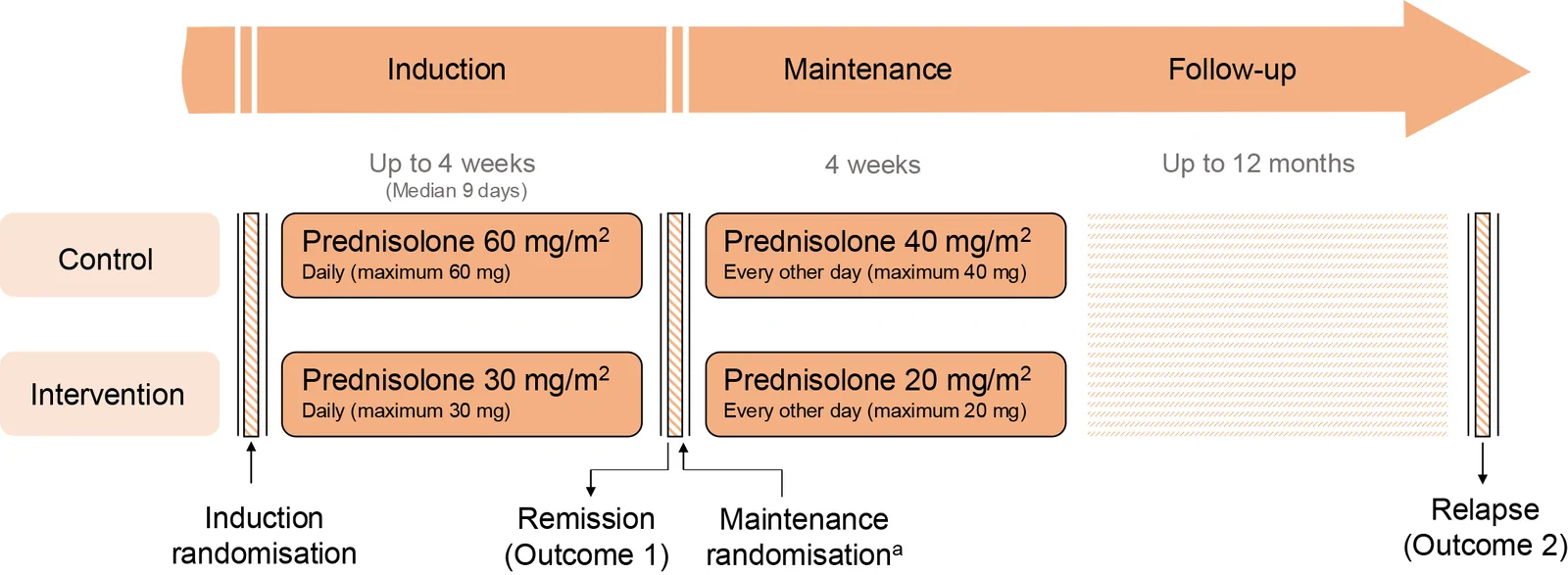
Schematic presentation of the OPEN trial design. ^a^If participants do not achieve remission, they then exit the trial at day 28. Participants may enter the trial at the maintenance phase after receiving nonrandomized, standard of care induction if entry to the trial would delay starting treatment of a relapse.

Randomization will occur twice, at the beginning of the induction and maintenance phases. The induction phase (median duration 9 days) relates to the time-to-remission outcome, and the 4-week maintenance phase relates to the time-to-relapse outcome. Participants will be followed for 9 months after commencement of the maintenance phase. When it is not possible for consent and allocation to occur at the commencement of induction (e.g., for some rural and remote families) participants will be allowed to enter the trial at the maintenance phase; this will be uncommon but is important to maintain equity of access.

## Methods

### Participants, Interventions, and Outcomes

#### Study Setting

All pediatric nephrology units in Australia (*n* = 7) and New Zealand (*n* = 1) will participate. These tertiary, academic hospitals treat all patients with steroid-dependent or frequently relapsing SSNS. They also treat most children with infrequently relapsing SSNS, either directly or in collaboration with general pediatricians at nontertiary centers.

#### Eligibility Criteria

##### Inclusion Criteria

Children from 1-year of age until their 16th birthday (inclusive), with a relapse of nephrotic syndrome, whose last relapse (or initial presentation if this is the first relapse) responded to prednisolone within 4 weeks.

Relapse of SSNS is defined as proteinuria of ≥ 3+ on urinalysis for ≥ 3 days, or ≥ 3+ on urinalysis with edema (≥ 3+ on urinalysis considered equivalent to a urine protein-to-creatinine of ≥ 200 mg/mmol). Remission (analogous to response) is defined as trace or negative proteinuria on urinalysis for 3 consecutive days (trace or negative on urinalysis considered equivalent to a urine protein-to-creatinine of ≤ 20 mg/mmol).^24^

##### Exclusion Criteria

Taking > 15 mg/m^2^ daily prednisolone at the time of recruitment or in the prior 2 weeks; familial nephrotic syndrome (a first-degree relative with nephrotic syndrome); for children who have undergone kidney biopsy, findings other than minimal change disease (with or without mesangial hypercellularity) or focal segmental glomerulosclerosis; children with a documented history of significant nonadherence or inability to perform daily urine dipsticks at home; children unable to take prednisolone capsules, even in dispersed form; or any condition resulting in life expectancy less than 1 year or that in the opinion of the investigator would present a serious safety risk to the participant.

#### Who Will Take Informed Consent?

Signed informed consent will be collected from a legal guardian of each participant by the site research nurse or investigator, after the provision of participant information sheets and any additional information. Assent will be obtained from participants assessed as capable of providing this by the research member performing the consent. Consent, and assent when applicable, will typically be performed in-person at the time of a routine clinic visit. This allows eligible children and guardians to decide about participation and ask relevant questions while they are well, as opposed to during an active relapse when there may be additional stresses upon the family. Rural participants may be consented by telehealth with signed documents returned by email.

#### Additional Consent Provisions for Collection and Use of Participant Data and Biological Specimens

No ancillary studies are currently planned and there will be no storage of biologic specimens for the purposes of this or other studies.

### Interventions

#### Explanation for the Choice of Comparators

In the induction phase, participants in the high-intensity group will receive 60 mg/m^2^ of prednisolone daily (maximum of 60 mg) until remission, compared with 30 mg/m^2^ of prednisolone daily (maximum of 30 mg) until remission in the low-intensity group. In the maintenance phase, participants in the high-intensity group will receive 40 mg/m^2^ (maximum of 40 mg) every other day for 4 weeks, compared with 20 mg/m^2^ (maximum of 20 mg) every other day for 4 weeks in the low-intensity group. The high-intensity doses correspond to the International Study of Kidney Disease in Children regimen previously described and are consistent with current guidelines from the International Pediatric Nephrology Association.^18^, ^24^ The low-intensity doses are half that of the high-intensity group but delivered over an identical duration and have been formulated based on existing data published in SSNS suggesting them to be safe and similarly effective to the high-intensity doses.^17^, ^19^, ^20^, ^21^, ^22^, ^23^, ^25^

#### Intervention Description

The study medication for both groups will be delivered by matching capsules containing either 5 mg (high-intensity) or 2.5 mg (low-intensity) of prednisolone powder with identical appearance and taste. Body surface area will be calculated using the DuBois formula and the dose rounded to the nearest capsule size.^26^, ^27^ The capsules may be opened and dispersed in liquid or a small amount of food (e.g., jam) for children who are unable to swallow them whole. Colored labels will be used to distinguish the bottles for induction and maintenance phases of treatment, but the containers and labels will be nonidentifiable with respect to the intervention. The study drug will be compounded, packed, and labeled at the central trial pharmacy. Specific resources regarding pill swallowing and dispersing capsules will be provided to participants.

#### Criteria for Discontinuing or Modifying Allocated Interventions

After 14 days, participants who are not in remission will be reviewed by the treating clinical team to ensure they are stable and can safely continue blinded treatment. After 28 days, participants who are not in remission will exit the trial and may be unblinded if deemed necessary by the treating clinical team. This is anticipated to be a rare event, given only children with steroid-sensitive disease are being enrolled and the intervention spans a single relapse only. During relapse, children with nephrotic syndrome may develop a wide range of complications. More common events, such as edema requiring diuretic or i.v. albumin administration, are expected to occur in both the trial groups. In addition, less common events, such as venous thromboembolism, are likely to be captured given the sample size. Participants may be unblinded and exit the trial at any point if the treating clinical team and family consider it necessary for participant safety; for example, in the presence of life-threatening complications and a delayed response to treatment for that participant.

#### Strategies to Improve Adherence to Interventions

The design of OPEN trial is pragmatic, with the goal of minimizing any additional burden of participation on families and clinical teams, having been developed in collaboration with consumers and clinicians at each of the participating sites. A consumer advisory board (CAB) has been formed, reporting directly to the Trial Steering Committee. The CAB will review all patient-facing material and make suggestions about additional resources as required. During the induction and maintenance phase, participants will regularly complete research electronic data capture (REDCap) forms, a failure to complete these may alert research staff to families at risk of reduced adherence providing the opportunity for early intervention to optimize participation. After delivery of the intervention, participants will be asked to return unused trial medication for verification that the intended per-protocol dose was delivered.

#### Relevant Concomitant Care Permitted or Prohibited During the Trial

There are no restrictions on the use or modification of non–corticosteroid medications or other concomitant care during the trial. Data on concomitant prescribed or nonprescribed medication used will be collected to allow for adjustment as potential confounders or effect modifiers in the statistical analysis.

#### Provisions for Post-Trial Care

The OPEN trial captures 1 relapse only. After completion of the trial, participants will resume standard care under direction of their treating clinical team. There is no provision for direct compensation to those who suffer harm from trial participation. Australia and New Zealand both have free, universal health care systems.

### Outcomes

The primary outcome for the induction phase is time-to-remission, with a noninferiority margin of 3 days (i.e., the trial is designed to exclude this difference between the groups, defined by the smallest clinically relevant difference, otherwise termed minimum clinically important difference). The primary outcome for the maintenance phase is time-to-next relapse, with a noninferiority margin of 30 days. The noninferiority margins were determined by discussion between investigators, including those with lived experience, and are consistent with the results from a recent Delphi consensus process conducted in Canada.^28^ Participants will be followed for 9 months after commencement of the maintenance phase. Relapse and remission are defined per the inclusion criteria. The effect size for both outcomes will be calculated using restricted mean survival time (RMST), a time-to-event metric that summarizes censored and noncensored participant outcome data within a clinically relevant time horizon. It is assumed that if 1 or both outcomes are noninferior then the reduction in corticosteroid exposure will result in fewer cumulative adverse events among children treated with low-intensity steroids, without a significant increase in the amount of time children are in relapse. The choice of time-to-relapse and time-to-remission as the primary trial outcomes is based on their being clearly defined and easily measured, important to children and their families, and with substantially more data available for the purposes of accurate sample size calculation and trial design.^29^

Adverse events known to be associated with corticosteroid use will be captured and analyzed as secondary outcomes. Potential corticosteroid adverse effects will be defined by investigator report, with guidance to include a new diagnosis of infection, abdominal pain, gastrointestinal bleeding, glaucoma, hypertension requiring antihypertensive therapy, hirsutism, excessive bruising, proximal muscle weakness, weight gain (when nonedematous), facial appearance (accumulation of facial fat), striae, sleep disturbance, mood disturbance, and disruptive behavior. Adverse effects of corticosteroid administration on quality of life, physical and psychosocial functioning will also be captured by questionnaire administration; Pediatric Quality of Life Inventory Generic Core Scales version 4.0 (PedsQL), Functional Assessment of Chronic Illness Therapy General Physical 5, and EuroQol 5-Dimension Youth version. The time to complete all surveys is estimated to be < 10 minutes (23-item PedsQL, single item Functional Assessment of Chronic Illness Therapy General Physical 5, and 5-item EuroQol 5-Dimension Youth version). PedsQL results will be aggregated by the physical and psychosocial health summary scores, in addition to the 4 functional scales (physical, emotional, social, and school), all being previously correlated with high-intensity prednisolone use among children with nephrotic syndrome.^25^, ^30^, ^31^

Tertiary outcomes will include a process evaluation conducted using a mixed methods approach to assess trial implementation (fidelity, quality, adaptation, and adoption), and mechanism (processes and pathways) underlying the intervention and a health economic evaluation. Trained researchers will conduct semistructured interviews with caregivers of children (both the groups) and health care professionals involved in the trial. Caregivers (*n* = 20) will be asked to participate in 1 interview when they exit the trial. Clinicians (*n* = 20) will participate in 1 interview after the trial concludes. Because this is a noninferiority trial, the health economic evaluation will normally consist of a cost minimization analysis. However, as reduced intensity prednisolone is hypothesized to result in better quality of life for children and to have long-term benefits not captured in the trial, the evaluation will include a within trial cost-effectiveness and cost utility (using EuroQol 5-Dimension Youth version data) analysis and a modeled evaluation over a longer time horizon. The within trial analysis will be from the perspective of the health care funder based on health care use estimated using trial data, hospital records, and by linked data from Medical Benefit Scheme and Pharmaceutical Benefit Scheme claims capturing outpatient costs within the Australian health care system. The modeled evaluation, which will take a broader societal perspective, using decision tree analysis and Markov model to estimate longer term outcomes. Model parameters will be informed by results from the trial, expert opinion, and the literature. The time horizon will be dependent on the availability and uncertainty of parameter estimates for longer term outcomes.

### Participant Timeline

Only children known to be steroid-sensitive during their previous relapse of nephrotic syndrome will be enrolled. In routine clinical care, families diagnose and treat uncomplicated relapses at home in collaboration with their treating team. To minimize participant burden, enrollment (written informed consent and assent) will occur on the same day as a routine clinical visit by an independent member of the research team. At that time, participants can elect to be randomized (both induction and maintenance phases) and collect their allocated treatment for each phase. At the time of relapse (treatment initiation), participants will contact their treating clinical or research team, who will verbally confirm participants continue to meet inclusion criteria and continue to agree to enter the active phase of the trial. Participants who have not had the opportunity to enroll before relapse may be included if they are able to provide consent and receive study drug in a reasonable timeframe (at treating teams’ discretion or within 48 hours). For rural patients or those with limited access to transport, virtual consent and couriering of study drug may be possible, but when not, participants can enter the trial at the maintenance phase after receiving nonrandomized induction treatment per usual clinical practice (for all participating units this is high-intensity prednisolone). The schedule of assessments before and after relapse is summarized below (Table 1):

**Table 1:** Schedule of enrollment, interventions, and assessment

Assessment procedure	Screening and consent	3 mo review	Start of relapse / induction	During induction	End Induction / start maintenance	End of Maintenance	Post-maintenance (3 mo)	Tertiary analysis
Eligibility criteria	x	x	X					
Informed consent	x							
Demographic	x	x						
Height/weight	x	x						
Medical history	x	x						
Randomization and allocation	x							
Dipstick test			x	x	x			
Documentation of relapse			x	x	x		x	
Patient diary				x	x			
Adverse events			x	x	x	x	x	
Concomitant medication	x		x	x	x	x		
PedsQL / FACT GP5 / EQ-5D-Y	x				x	x		
Trial medication compliance				x	x	x		
Hospital service utilization								x
MBS, PBS data								x

MBS, medicare benefits schedule; mo, month(s); PBS, pharmaceutical benefits scheme.

### Sample Size

The estimated number of participants is 330, allowing for 10% dropout. This is based upon the time-to-relapse for the maintenance phase, which requires a larger number of participants than the induction phase. All sample size calculations were performed using R software, version 4.0 (R Core Team, Vienna, Austria) via simulation. Power for both phases was calculated assuming a true difference in RMST of 0 days between the groups (identical event-time distributions, equivalent to a hazard ratio of 1), consistent with the existing comparative data,^19^, ^20^, ^21^, ^22^, ^23^ and defined as the proportion of 1000 simulations in which the 1-sided 97.5% confidence bound for the difference in RMST excluded the noninferiority margin.

The median time-to-relapse after maintenance was estimated to be 100 days based on the most similar contemporary trial cohort, short course daily prednisolone therapy during an upper respiratory tract infection in children with relapsing SSNS (Prednisolone in Nephrotic Syndrome 2).^32^ To model this survival distribution, a piecewise exponential survival model was used, assuming an event rate of 0.21 until 7 months, followed by an event rate of 0.03 in both the groups. Under these assumptions, 71% of patients would have relapsed by 6 months after maintenance and 80% by 12 months. A noninferiority margin of 30 days was specified, with the RMST estimated over a 9-month time horizon. Based on these parameters, a total sample size of 300 participants (150 per group) provides 80% power to demonstrate noninferiority at a 1-sided significance level of 0.025, assuming a follow-up of 9 months. Sensitivity simulations confirmed that the most important factor impacting sample size was the follow-up period in relation to the noninferiority margin, which was determined based on a prespecified acceptable clinical margin.^33^, ^34^, ^35^

For the induction phase, the remission times were assumed to follow an exponential distribution, with a rate of 0.075 per day, that results in a median time to remission of 9 days, with all patients achieving remission within the 28-day induction window.^36^, ^37^ A clinically relevant noninferiority margin of 3 days was prespecified. Noninferiority was assessed using the difference in RMST over a 14 day horizon. Given the planned sample size of 300 participants entering the maintenance phase, the study has greater than 99% power to demonstrate noninferiority at a 1-sided significance level of 0.025.

### Recruitment

Engagement with consumers before and during the development of the protocol indicates that families are highly engaged with the topic of this trial. This has resulted in a pragmatic protocol designed in conjunction with the trial’s CAB to minimize inconvenience and disruption to participants and families. The CAB will also advise on communication strategies and develop additional patient-facing resources. Recruitment will also be monitored throughout the period of trial conduct by the Trial Steering Committee and Data Monitoring Committee (DMC).

### Assignment of Interventions: Allocation

#### Sequence Generation

The allocation sequence will be generated by computer randomization using randomly permuted block sizes of varying length, stratified by site. The stratification removes the possibility of bias due to differences in concomitant care and additional follow-up beyond the specified minimum frequencies between centers and facilitates distribution of study medication.

#### Concealment Mechanism

Allocation is concealed from trial participants, investigators, site staff, care providers, and outcome assessors throughout the trial period. Allocation is known to the core pharmacy and in coded form to the core administrative team, who do not have participant contact and are not involved in data collection or outcome assessment, and will advise site pharmacies in case of additional medication supply requirements.

#### Implementation

The allocation sequence will be generated and held by the central pharmacy, who are not involved in patient enrollment. Participants will be enrolled by the local research team at which they will receive a sequential study identifier. The study medication will then be allocated according to blinded study bottles labeled only by study identifier and study phase (induction or maintenance).

### Assignment of Interventions: Blinding

#### Who Will Be Blinded

The study drug packaging and distribution will maintain blinding of the site pharmacy, trial participants, and clinical and local research teams. Neither the Trial Steering Committee, CAB, or Trial Management Team will have any access to the allocation sequence or allocation. The final analysis will be performed by a blinded statistician. If requested by the DMC, unblinded reports requiring knowledge of treatment allocation will be prepared by an independent statistician.

#### Procedure for Unblinding if Needed

The allocation sequence will be held by the central pharmacy, which will be contacted if unblinding is required. Site Principal Investigators may request unblinding for children who experience a serious adverse event (SAE) when it is justified on the basis that unblinding will directly impact patient care. Children who do not enter remission after 28 days in the induction phase will exit the trial as described and be unblinded if required to guide further treatment.

### Data Collection and Management

#### Plans for Assessment and Collection of Outcomes

Time-to-remission will be captured by daily REDCap forms completed by participants during the induction phase. Time-to-relapse will be captured by REDCap forms completed weekly by participants during the maintenance phase and then at relapse thereafter. The local research team will be asked to confirm outcome events (remission by photograph of the urine dipstick at completion of induction and relapse by report or prescription of restarting prednisolone) and relapse status at least every 3 months until the 9 months of follow-up is completed.

#### Plans to Promote Participant Retention and Complete Follow-up

Site- and trial-level strategies will be employed to promote participant retention and complete follow-up. Treating clinical teams will be encouraged to engage with participants about their experience of trial participation and promote the importance of complete follow-up to accurate results. Site Principal Investigators will provide presentations to treating clinical teams outlining the key components of the trial protocol and provide regular updates on trial progress to maintain clinician engagement. Trial-level strategies will include a 6-monthly clinical trial newsletter outlining progress, highlighting the contribution of specific sites, providing questions and answers from the CAB, and providing contact details for Site Principal Investigators and the Sponsor. A trial website will be established that includes these newsletters and further information about the trial, including short videos presenting information about the trial in an accessible format for families.

Participants who withdraw from the trial will have the option to allow the retention of preexisting data for analysis, in which case their participation will contribute until censoring at the point of exit.

In the absence of active withdrawal, if participants are not contactable to the research team for follow-up after maintenance to confirm either primary outcome, then the clinical team will be contacted to confirm the outcome status.

#### Data Management

The trial Data Management Plan will be approved by the Trial Management Team and Trial Steering Committee. Sites will be responsible for data collection and entry of staff-administered processes, into a custom REDCap database hosted by Flinders University. Database training for site staff will form part of their induction and delegation process. Data quality checks such as validation formats, use of multiple choice or restricted answer formats rather than free text and expected or acceptable ranges will be built into the database when possible. The data manager in the core administrative team will run periodic quality checks throughout the period of trial conduct and issue queries to sites for verification, clarification or amendments as required. Such changes are logged using the inbuilt audit trail functions in REDCap.

Participant or caregiver diaries and self or caregiver-reported outcomes will be entered directly into the trial’s online database using individual custom links, unless participant or caregivers request use of paper forms, in which case content of the paper forms will mimic the electronic form and will be returned to and entered into the electronic database by site staff as soon as practicable after collection.

Site copies of electronic documents and an electronic investigator site file will be housed on a secure institutional server at each location with access restricted to authorized personnel. Any paper files and forms will be stored in a secure physical location at the site (e.g., locked filing cabinet or swipe card accessible office) and then archived in an institutional archive consistent with the site’s governance arrangements after study completion, for the duration specified in the ethics approval. Data on health service use that is not provided by the hospital is supplied by Services Australia under project-specific approval consistent with consent from individual participants.

#### Confidentiality

The database used will be encrypted and backed up securely on an institutional server, and accessible using multifactor authentication to delegated individuals only. Site staff will only have access to enter or amend data from their own site using User Rights and Data Access Group delegation functions in REDCap as appropriate, with no ability to extract or export data. Upon completion of data collection and addressing of queries, the electronic database will be locked to site staff and a cleaned and deidentified copy of the final dataset will be securely transferred to the study statistician for analysis, to be stored on an institutional server with appropriate backup and accessible only to suitably qualified and delegated personnel. Linked data provided by Services Australia is de-identified and uses an individual project ID to enable analysis by the project health economists.

#### Plans for Collection, Laboratory Evaluation and Storage of Biological Specimens for Genetic or Molecular Analysis in this Trial or Future use

No additional laboratory evaluations beyond standard of care will be collected. For most children no samples are required, but these may be requested by the treating team per standard of care (e.g., to confirm nephrotic range proteinuria if there is uncertainty about a dipstick result, or to diagnose complications such as acute kidney injury in patients with nephrosis and intravascular volume depletion). Any samples collected as part of usual care will be processed and analyzed according to usual clinical pathways at each site by National Association of Testing Authorities accredited laboratories.

### Statistical Methods

#### Statistical Methods for Primary and Secondary Outcomes

The primary outcomes will be analyzed using RMST among all patients who receive at least 1 dose of the study medication (modified intention-to-treat population). Both primary outcomes are noninferiority analyses, evaluated with a 1-sided alpha of 0.025 (equivalent to a 2-sided alpha of 0.05 used in superiority trials). No alpha adjustment was applied, treating the phases as sequential outcomes for those who enter at the induction phase (gatekeeping procedure).^38^ The noninferiority margin is set at 3 days for time to remission within a 28-day time horizon, and 30 days for time to relapse within a 270-day time horizon. The RMST in each arm will be estimated nonparametrically as the area under the Kaplan-Meier curve to the prespecified horizon, and the groups compared by the difference in RMST (low-intensity − high-intensity) with a 2-sided 95% confidence interval.^33^, ^34^, ^35^ Noninferiority will be concluded if the confidence bound in the direction of inferiority excludes the margin: an upper bound below 3 days for time-to-remission, and a lower bound above −30 days for time-to-relapse. Participants censored before the horizon (for example, withdrawal of consent or loss to follow-up) contribute follow-up until censoring through the Kaplan-Meier risk sets, under the assumption of noninformative censoring, whereas participants event-free at the horizon contribute its full value, avoiding extrapolation beyond the observed data. Secondary outcomes will compare the number of children who fail to enter remission during the induction phase, number of unscheduled general practitioner or hospital visits, admissions to hospital, and the total cumulative incidence of SAE and adverse events between the groups. Continuous patient reported outcome scores will be standardized for comparison, with an effect size 0.2 (small), 0.5 (medium), and 0.8 (large). Statistical significance will be determined by *t* test (or Mann-Whitney U test if the distribution is skewed) and by chi-square test for difference in proportions. A 2-sided alpha of 0.05 will be used for secondary outcomes. This is because the low-intensity group may have a greater number or severity of complications due a delayed trajectory towards remission, or conversely the low-intensity group may experience fewer complications due to a lower corticosteroid dose if the primary outcomes are not significantly different, as hypothesized. This contrasts with the primary outcome whereby the only plausible direction of effect is towards an increased time-to-remission or time-to-relapse in the low-intensity group. A complete statistical analysis plan will be prepared and approved by the Trial Steering Committee before any analysis.

#### Interim Analyses

For safety reasons, a futility analysis will be conducted at the midpoint of recruitment. Given the noninferiority design, futility would be indicated by superiority of the high-intensity group and in such a scenario continuing randomization would be considered unethical. Therefore, if the conditional power falls below 0.1 at midpoint of the sample size, a superiority test will be conducted for the high-intensity (control) group. If the high-intensity group demonstrates superiority at a 2-sided alpha of 0.05, randomization to the low-intensity group for that phase of the trial will be discontinued. If the superiority test does not achieve statistical significance, the DMC will review the results and make a recommendation to the Trial Steering Committee on whether to continue with randomization. The final decision for continuation is the responsibility of the Trial Steering Committee.

#### Methods for Additional Analyses (e.g., Subgroup Analyses)

Adjusted analyses will be performed to account for chance variation in relevant confounders, including age, proportion of children receiving concurrent noncorticosteroid immunosuppression, and ethnicity. Sensitivity analyses using interaction terms for age, concurrent noncorticosteroid immunosuppression, and ethnicity will be individually performed to detect effect modification based on *a priori* plausibility. The possibility of effect modification in the maintenance phase by induction allocation will also be tested. Sensitivity analyses will include a per protocol dataset consisting of only participants who report taking ≥ 75% of the study medication.

#### Methods in Analysis to Handle Protocol Nonadherence and any Statistical Methods to Handle Missing Data

All primary analyses will be reported as modified intention-to-treat. If the proportion of missing data for any covariate is ≥ 10% then multiple imputation by chained equations will be used to account for missingness. If present, missing outcome data will be censored at the last observed status (unlikely to be substantial given the need for routine clinical follow-up).

#### Plans to Give Access to the Full Protocol, Participant Level-Data and Statistical Code

The full protocol and statistical analysis plan will be made available upon request. Aggregate or participant-level data may be made available for secondary use upon appropriate request to the sponsor, after approval of the proposed activity by the Trial Steering Committee if deemed consistent with the trial’s Data Sharing Plan and with relevant ethics approvals in place. Statistical code will be available upon request.

### Oversight and Monitoring

#### Composition of the Coordinating Centre and Trial Steering Committee

Trial governance will occur within the following framework (Figure 2).

**Figure 2:**
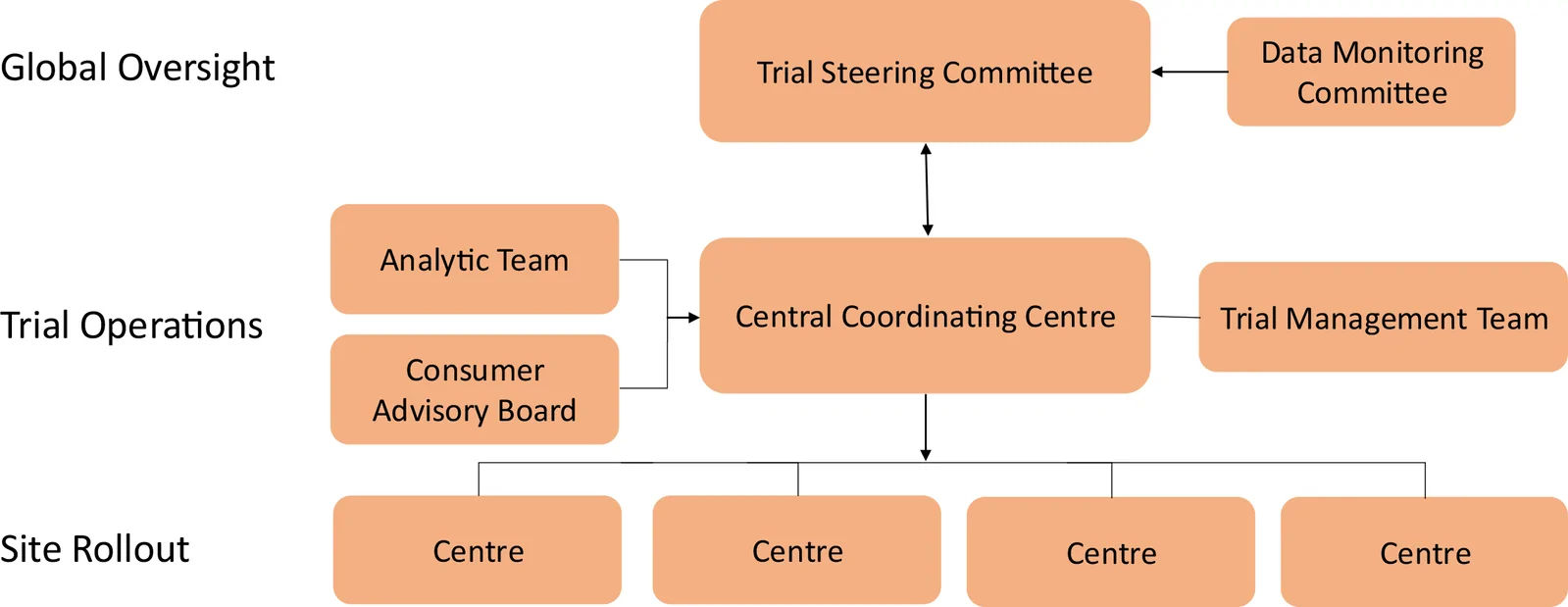
Trial governance.

The Trial Steering Committee comprises 24 members, including 3 people with lived experience of nephrotic syndrome. The Trial Steering Committee will meet monthly until all sites are active and then every second month thereafter. The Central Coordinating Centre is the Flinders University Clinical Trials Unit. The Trial Management Team will meet every second week and include representatives from the Central Coordinating Centre and Coordinating Principal Investigators. The analytic team consisted of 2 biostatisticians, qualitative experts, and a health economist. The 8-member CAB will meet monthly during trial development and then every third month once all sites are active. The CAB will review all participant facing material, the trial protocol, and the CAB Chair will sit on the Trial Steering Committee. Each center will be represented on the Trial Steering Committee by at least 1 member, and at the site level every center will have at least 1 other investigator as backup for trial operations in case the Site Investigator is unavailable.

#### Composition of the Data Monitoring Committee, its Role and Reporting Structure

A 4-member, independent DMC will be formed and will operate under a predefined terms of reference (available upon request), agreed to by the Chair and ratified by the Trial Steering Committee. The DMC Chair will be a senior nephrologist, and the other 3 members a biostatistician and senior nephrologists with appropriate research experience. The DMC will meet before recruitment of the first patient, and thereafter as often as they deem necessary. An interim analysis will be performed at the mid-point of the trial as described. Safety reports detailing adverse effects and SAE will be prepared for the DMC for review after 75 and 200 participants have completed 3-months of follow-up, unblinded to treatment allocation if requested by the DMC Chair. The DMC will monitor recruitment throughout the study regarding feasibility to reach the calculated sample size.

#### Adverse Event Reporting and Harms

Adverse events will be recorded by participants during the induction and maintenance phase at a frequency of daily and weekly, respectively. Specific events related to corticosteroid administration or nephrosis, which may overlap, will be listed to encourage complete reporting. These include infection, abdominal pain, gastrointestinal bleeding, glaucoma, hypertension requiring antihypertensive therapy, hirsutism, excessive bruising, proximal muscle weakness, weight gain, altered facial appearance, striae, sleep disturbance, mood disturbance, or disruptive behaviour. SAE will be collected from local clinical and research teams, defined as any death or event that is life-threatening, requires inpatient hospitalization or prolongs an existing hospitalization, results in persistent or significant disability or incapacity at any time if possibly related to trial participation. SAE will be reported to the Trial Management Team within 24 hours of the local research team becoming aware. The Trial Management Team will then compile a report for the DMC and Human Research Ethics Committee within the next 72 hours. All documented adverse events will be reported at the preferred term level according to standardized (MedDRA) terminology.

#### Frequency and Plans for Auditing Trial Conduct

Project managers from the core administrative team at Flinders University will conduct monitoring on behalf of the Sponsor and independent of investigators, based on documented risk-based monitoring plan, under which early trial activity at each site will be prioritized for monitoring and then again at study close-out, with additional monitoring in the interim based on assessed risk. Trial conduct may also be audited by ethics committees and the funder Medical Research Future Funds or National Health and Medical Research Council at any point.

#### Plans for Communicating Important Protocol Amendments to Relevant Parties (e.g., Tial participants and Ethical Committees)

Amendments will be submitted to the Women’s and Children’s Hospital Human Research Ethics Committee, followed by the Human Research Ethics Committee at participating centers for acceptance under the Australian National Mutual Acceptance scheme. The CAB will review any amendments, and significant changes will be communicated to participants.

#### Dissemination Plans

An open-access, peer-reviewed manuscript will be published reporting the trial results. Additional manuscripts will be published containing further detail on the qualitative outcome data and process evaluation, and health economic analysis. Links to these publications, as well as lay summaries written in plain English and reviewed by the CAB will be provided directly to participants via the trial newsletter and website. The Australian New Zealand Clinical Trials Registry will be updated when enrollment has closed and then when the results have been published.

## Discussion

The OPEN trial will definitively determine if low-intensity corticosteroid dosing for the induction and maintenance of remission in childhood SSNS is safe and effective, directly addressing a key priority identified by children and families with lived experience of SSNS.^39^, ^40^ If noninferiority of the low-intensity arm compared with the high-intensity arm is demonstrated, then adopting the low-intensity arm in clinical care would halve the cumulative corticosteroid burden of children with SSNS. Importantly, the OPEN trial will enroll children regardless of noncorticosteroid immunosuppressive use. Half of children with SSNS are prescribed a noncorticosteroid immunosuppressive agent at some point, typically for frequently relapsing or steroid-dependent disease, and it is this group with the highest relapse frequency who stand to benefit most from a reduction in cumulative corticosteroid exposure.^5^, ^11^

The main risk to successful trial completion is enrollment. The sample size is large relative to the Australian and New Zealand population and will require enrollment of up to 50% of children with SSNS, based on feasibility audits and historical data regarding the incidence of nephrotic syndrome in Australia.^5^ To optimize enrollment, a pragmatic trial design has been developed in collaboration with consumers and all pediatric nephrology units in both countries with the aim of lowering barriers to trial participation. Mitigation strategies for slow enrollment include extending the duration of the trial or adding international sites. The study medication is planned to be delivered as capsules, which can be opened and dispersed in liquid or another substance (e.g., teaspoon of jam) for younger children. This differs from routine practice in Australia and New Zealand but is similar to the approach used in recent trials such as Prednisolone in Nephrotic Syndrome 2, which proved to be well tolerated and effective.^32^

The double randomization design of the OPEN trial aligns with routine clinical care by splitting the trial into induction and maintenance phases. From a causal perspective, this assumes no carry-over effect from induction treatment on the response to maintenance allocation. The median time-to-remission is estimated to be 9-days, compared with the fixed-period 28-day maintenance phase. The median time-to-relapse is estimated to be 100 days. It seems unlikely that a relatively short period of induction treatment will significantly modify the effect of the maintenance treatment on the distal outcome event (time-to-relapse). Regardless, the possibility of effect modification by induction allocation will be tested as a sensitivity analysis. If an interaction was found, then this would be important data to guide future treatment strategies and trial design. One important advantage of separating the induction and maintenance phases is to allow children who cannot receive induction medication in time to still participate in the trial. This is important for equity of access among participants living in rural and remote regions. Australia has an urbanized population but large land mass and low population density outside of major cities. Therefore, relatively few participants (≤ 10%) are expected to enter the trial at the maintenance phase. Given the variance of time-to-relapse relative to the noninferiority margin is much greater than for time-to-remission, the sample size estimate is driven by the maintenance phase and there will still be a power of ≥ 90% for the induction phase at the predesignated noninferiority margin (the induction phase is adequately powered to tolerate the loss of ≥ 25% participants).

There are few patient-reported outcome measures available for children with nephrotic syndrome and the measures to be used in this trial have not been specifically designed for or validated among children with SSNS. PedsQL is the most frequently used tool to measure quality of life among studies enrolling children with SSNS, and the Generic Core Scales include questions directly addressing many of the key side effects of corticosteroid administration.^41^ PedsQL is also one of the most widely validated instruments used to assess the quality of life of children with chronic disease generally, including across geographic settings and among culturally and linguistically diverse populations.^42^ The choice of primary and secondary outcomes being measured in the OPEN trial is informed by the Standardized Outcomes in Nephrology initiative–Children and Adolescents and Glomerular Disease core outcomes.^43^, ^44^ The OPEN trial includes process evaluation as a tertiary outcome assessing trial conduct to inform future trial design, and identify enablers and barriers to implementation of the results.

Recent efforts to identify the causative antibodies underlying SSNS are partly motivated by the possibility that these might inform more personalized treatment approaches, as has occurred with membranous nephropathy.^7^, ^8^, ^45^ Such advances may reduce the overall relapse frequency of children with SSNS. Regardless, it is expected that SSNS will remain a relapsing-remitting condition requiring repeated courses of corticosteroids. It is also expected to be several years before such tests are sufficiently developed and available to inform clinical practice.

The OPEN trial is a large, randomized, controlled trial that will determine if low-intensity corticosteroid treatment for relapsed SSNS is noninferior to the current guideline-recommended standard of care, high-intensity corticosteroid treatment. It will test both time-to-remission and time-to-relapse via separate induction and maintenance randomizations. The protocol specifically includes children receiving concurrent noncorticosteroid treatments, a large group for whom scarce data currently exist but who have the highest cumulative corticosteroid exposure. Enrollment is anticipated to be the greatest barrier to successful completion of the trial. However, the sample size is achievable based on the broad, binational collaboration among a highly motivated group of clinicians and consumers.

## Trial Status

Protocol Number 1, Version 2.0.

Date: 26/09/2024.

Recruitment is intended to commence in December 2025 and end May 2028.

## Disclosure

The authors declare that they have no competing interests beyond the scope of their own normal employment.

## Ethics Approval and Consent to Participate

Ethics approval was obtained from the Women’s and Children’s HREC (2024/HRE00131) according to the National Mutual Acceptance Scheme, and the New Zealand Health and Disability Ethics (HDEC) Committee (2025 FULL 20608). Written, informed consent will be obtained from a legal guardian of all participants and assent will be obtained from children, appropriate to their level of comprehension.

## Consent for Publication

The participant information and consent forms include consent for publication. A model consent form is available on request.

## References

[1] Veltkamp F., Rensma L.R., Bouts A.H.M., LEARNS Consortium (2021). Incidence and relapse of idiopathic nephrotic syndrome: meta-analysis. Pediatrics.

[2] Vivarelli M., Gibson K., Sinha A., Boyer O. (2023). Childhood nephrotic syndrome. Lancet.

[3] Arneil G. (1961). 164 children with nephrosis. Lancet.

[4] International Study of Kidney Disease in Children (1981). The primary nephrotic syndrome in children. Identification of patients with minimal change nephrotic syndrome from initial response to prednisone. A report of the International Study of Kidney Disease in Children. J Pediatr.

[5] Sureshkumar P., Hodson E.M., Willis N.S., Barzi F., Craig J.C. (2014). Predictors of remission and relapse in idiopathic nephrotic syndrome: a prospective cohort study. Pediatr Nephrol.

[6] Kim L.H., Catapang M., Polderman N. (2024). Outcomes using a standardized provincial childhood nephrotic syndrome clinical pathway. Can J Kidney Health Dis.

[7] Raglianti V., Angelotti M.L., Cirillo L. (2024). Anti-slit diaphragm antibodies on kidney biopsy identify pediatric patients with steroid-resistant nephrotic syndrome responsive to second-line immunosuppressants. Kidney Int.

[8] Hengel F.E., Dehde S., Lassé M. (2024). Autoantibodies targeting Nephrin in podocytopathies. N Engl J Med.

[9] Arneil G.C. (1971). The nephrotic syndrome. Pediatr Clin N Am.

[10] Kikunaga K., Ishikura K., Terano C. (2017). High incidence of idiopathic nephrotic syndrome in East Asian children: a nationwide survey in Japan (JP-SHINE study). Clin Exp Nephrol.

[11] Tarshish P., Tobin J., Bernstein J. (1997). CME. Prognostic change study significance nephrotic disease of the early report in children course of minimal syndrome: report of the international study of kidney disease in children. J Am Soc Nephrol.

[12] Aljebab F., Choonara I., Conroy S. (2016). Systematic review of the toxicity of short-course oral corticosteroids in children. Arch Dis Child.

[13] Selewski D.T., Troost J.P., Massengill S.F. (2015). The impact of disease duration on quality of life in children with nephrotic syndrome: a Midwest Pediatric Nephrology Consortium study. Pediatr Nephrol.

[14] Lee J.M., Kronbichler A., Shin J.I., Oh J. (2020). Review on long-term non-renal complications of childhood nephrotic syndrome. Acta Paediatr.

[15] Bharati J., Tiewsoh K., Dawman L. (2023). Long-term complications in patients with childhood-onset nephrotic syndrome. Pediatr Nephrol.

[16] Larkins N.G., Hahn D., Liu I.D., Willis N.S., Craig J.C., Hodson E.M. (2024). Non-corticosteroid immunosuppressive medications for steroid-sensitive nephrotic syndrome in children. Cochrane Database Syst Rev.

[17] Hahn D., Samuel S.M., Willis N.S., Craig J.C., Hodson E.M. (2024). Corticosteroid therapy for nephrotic syndrome in children. Cochrane Database Syst Rev.

[18] Primack W.A., Chevalier R.L., Friedman A. (2023). The first randomized controlled trial in pediatric nephrology: the history of the International Study of Kidney Disease in Children (ISKDC). Pediatr Nephrol.

[19] Sheikh S., Mishra K., Kumar M. (2021). Low-dose versus conventional-dose prednisolone for nephrotic syndrome relapses: a randomized controlled non-inferiority trial. Pediatr Nephrol.

[20] Borovitz Y., Alfandary H., Haskin O. (2020). Lower prednisone dosing for steroid-sensitive nephrotic syndrome relapse: a prospective randomized pilot study. Eur J Pediatr.

[21] Tu J., Chen C.Y., Geng H.Y. (2022). Clinical assessment of moderate-dose glucocorticoid in the treatment of recurrence of primary nephrotic syndrome in children: a prospective randomized controlled trial. Zhong Guo Dang Dai Er Ke Za Zhi.

[22] Kainth D., Hari P., Sinha A., Pandey S., Bagga A. (2021). Short-duration prednisolone in children with nephrotic syndrome relapse: a noninferiority randomized controlled trial. Clin J Am Soc Nephrol.

[23] Mantan M., Kansal A., Swarnim S. (2022). Effectiveness of a low dose prednisolone regimen for treatment of relapses in children with steroid sensitive nephrotic syndrome. Indian J Nephrol.

[24] Trautmann A., Boyer O., Hodson E. (2023). IPNA clinical practice recommendations for the diagnosis and management of children with steroid-sensitive nephrotic syndrome. Pediatr Nephrol.

[25] Raja K., Parikh A., Webb H., Hothi D. (2017). Use of a low-dose prednisolone regimen to treat a relapse of steroid-sensitive nephrotic syndrome in children. Pediatr Nephrol.

[26] Sigurdsson T.S., Lindberg L. (2020). Six commonly used empirical body surface area formulas disagreed in young children undergoing corrective heart surgery. Acta Paediatr.

[27] DuBois D. (1916). A formula to estimate the approximate surface area if height and weight be known. Arch Intern Med.

[28] Robinson C.H., Carter S., Aman N. (2025). Establishing core outcomes and minimal clinically important differences for childhood steroid sensitive nephrotic syndrome clinical trials: results from a Delphi consensus process. Pediatr Nephrol.

[29] Samuel S.M., Takano T., Scott S. (2017). Setting new directions for research in childhood nephrotic syndrome: results from a National workshop. Can J Kidney Health Dis.

[30] Varni J.W., Seid M., Kurtin P.S., Peds Q.L. (2001). PedsQL 4.0: reliability and validity of the Pediatric Quality of Life Inventory version 4.0 generic core scales in healthy and patient populations. Med Care.

[31] Varni J.W., Seid M., Rode C.A. (1999). The PedsQL™: measurement model for the pediatric quality of life inventory. Med Care.

[32] Christian M.T., Webb N.J.A., Mehta S. (2022). Evaluation of daily low-dose prednisolone during upper respiratory tract infection to prevent relapse in children with relapsing steroid-sensitive nephrotic syndrome: the PREDNOS 2 randomized clinical trial. JAMA Pediatr.

[33] Charu V., Tian L., Kurella Tamura M., Montez-Rath M.E. (2024). Using restricted mean survival time to improve interpretability of time-to-event data analysis. Clin J Am Soc Nephrol.

[34] Royston P., Parmar M.K. (2013). Restricted mean survival time: an alternative to the hazard ratio for the design and analysis of randomized trials with a time-to-event outcome. BMC Med Res Methodol.

[35] Eaton A., Therneau T., Le-Rademacher J. (2020). Designing clinical trials with (restricted) mean survival time endpoint: practical considerations. Clin Trials.

[36] Dossier C., Delbet J.D., Boyer O. (2019). Five-year outcome of children with idiopathic nephrotic syndrome: the NEPHROVIR population-based cohort study. Pediatr Nephrol.

[37] Nardini B., La Scola C., Corrado C. (2025). Time to remission in childhood steroid sensitive nephrotic syndrome: a change in perspective. Eur J Pediatr.

[38] Dmitrienko A., Tamhane A.C. (2007). Gatekeeping procedures with clinical trial applications. Pharm Stat.

[39] Robinson C.H., Aman N., Banh T. (2025). Comparative efficacy of nonsteroid immunosuppressive medications in childhood nephrotic syndrome. JAMA Pediatr.

[40] Okpere A., Samuel S., King-Shier K., Hamiwka L., Elliott M. (2025). Patient and caregiver perspectives on treatment strategies and care priorities of childhood steroid-sensitive nephrotic syndrome: a qualitative study. BMJ Open.

[41] Aronu A.E., Uwaezuoke S.N., Muoneke U.V. (2021). Reliability of generic quality-of-life instruments in assessing health-related quality of life among children and adolescents with idiopathic nephrotic syndrome: a systematic review. Health Qual Life Outcomes.

[42] Varni J.W., Burwinkle T.M., Seid M. (2005). The PedsQL™ as a pediatric patient-reported outcome: reliability and validity of the PedsQL™ Measurement Model in 25,000 children. Expert Rev Pharmacoecon Outcomes Res.

[43] Carter S.A., Lightstone L., Cattran D. (2022). A core outcome set for trials in glomerular disease: a report of the standardized outcomes in nephrology–glomerular disease (SONG-GD) stakeholder workshops. Clin J Am Soc Nephrol.

[44] Hanson C.S., Craig J.C., Logeman C. (2020). Establishing core outcome domains in pediatric kidney disease: report of the Standardized Outcomes in Nephrology—children and Adolescents (SONG-KIDS) consensus workshops. Kidney Int.

[45] Ronco P., Beck L., Debiec H. (2021). Membranous nephropathy. Nat Rev Dis Prim.

